# Patient Engagement and Provider Effectiveness of a Novel Sleep Telehealth Platform and Remote Monitoring Assessment in the US Military: Pilot Study Providing Evidence-Based Sleep Treatment Recommendations

**DOI:** 10.2196/47356

**Published:** 2023-11-16

**Authors:** Emerson M Wickwire, Jacob Collen, Vincent F Capaldi, Scott G Williams, Samson Z Assefa, Julianna P Adornetti, Kathleen Huang, Janet M Venezia, Rachell L Jones, Christine W Johnston, Connie Thomas, Mary Ann Thomas, Charles Mounts, Christopher L Drake, Michael S Businelle, Michael A Grandner, Rachel Manber, Jennifer S Albrecht

**Affiliations:** 1 Sleep Disorders Center, Division of Pulmonary and Critical Care Medicine Department of Medicine University of Maryland School of Medicine Baltimore, MD United States; 2 Sleep Disorders Center Walter Reed National Military Medical Center Bethesda, MD United States; 3 Department of Medicine Uniformed Services University of the Health Sciences Bethesda, MD United States; 4 Center for Military Psychiatry and Neuroscience Walter Reed Army Institute of Research Silver Spring, MD United States; 5 Sleep Disorders Center Fort Belvoir Community Hospital Fort Belvoir, VA United States; 6 Division of Pulmonary and Critical Care Medicine Department of Medicine University of Maryland School of Medicine Baltimore, MD United States; 7 Center for Military Psychiatry and Neuroscience Behavioral Biology Branch Walter Reed Army Institute of Research Silver Spring, MD United States; 8 Sleep Research Center Henry Ford Hospital Detroit, MI United States; 9 TSET Health Promotion Research Center Stephenson Cancer Center Oklahoma City, OK United States; 10 Department of Psychiatry University of Arizona College of Medicine Tucson, AZ United States; 11 Department of Psychiatry and Behavioral Sciences School of Medicine Stanford University Palo Alto, CA United States; 12 Department of Epidemiology and Public Health University of Maryland School of Medicine Baltimore, MD United States

**Keywords:** sleep, sleep disorders, insomnia, obstructive sleep apnea, telehealth, remote monitoring, monitoring, patient engagement, sleep, effectiveness, effective care, behavioral, care, application, wearables

## Abstract

**Background:**

Sleep problems are common and costly in the US military. Yet, within the military health system, there is a gross shortage of trained specialist providers to address sleep problems. As a result, demand for sleep medicine care far exceeds the available supply. Telehealth including telemedicine, mobile health, and wearables represents promising approaches to increase access to high-quality and cost-effective care.

**Objective:**

The purpose of this study was to evaluate patient engagement and provider perceived effectiveness of a novel sleep telehealth platform and remote monitoring assessment in the US military. The platform includes a desktop web portal, native mobile app, and integrated wearable sensors (ie, a commercial off-the-shelf sleep tracker [Fitbit]). The goal of the remote monitoring assessment was to provide evidence-based sleep treatment recommendations to patients and providers.

**Methods:**

Patients with sleep problems were recruited from the Internal Medicine clinic at Walter Reed National Military Medical Center. Patients completed intensive remote monitoring assessments over 10 days (including a baseline intake questionnaire, daily sleep diaries, and 2 daily symptom surveys), and wore a Fitbit sleep tracker. Following the remote monitoring period, patients received assessment results and personalized sleep education in the mobile app. In parallel, providers received a provisional patient assessment report in an editable electronic document format. Patient engagement was assessed via behavioral adherence metrics that were determined a priori. Patients also completed a brief survey regarding ease of completion. Provider effectiveness was assessed via an anonymous survey.

**Results:**

In total, 35 patients with sleep problems participated in the study. There were no dropouts. Results indicated a high level of engagement with the sleep telehealth platform, with all participants having completed the baseline remote assessment, reviewed their personalized sleep assessment report, and completed the satisfaction survey. Patients completed 95.1% of sleep diaries and 95.3% of symptom surveys over 10 days. Patients reported high levels of satisfaction with most aspects of the remote monitoring assessment. In total, 24 primary care providers also participated and completed the anonymous survey. The results indicate high levels of perceived effectiveness and identified important potential benefits from adopting a sleep telehealth approach throughout the US military health care system.

**Conclusions:**

Military patients with sleep problems and military primary care providers demonstrated high levels of engagement and satisfaction with a novel sleep telehealth platform and remote monitoring assessment. Sleep telehealth approaches represent a potential pathway to increase access to evidence-based sleep medicine care in the US military. Further evaluation is warranted.

## Introduction

In part due to an unrelenting tempo and nontraditional work hours, insufficient and disturbed sleep are common and costly within the US military. Relative to civilians, active-duty military personnel are far less likely to have an adequate sleep duration [1-4]. Clinical sleep disorders such as insomnia, obstructive sleep apnea, shift work disorder, nightmare disorder, and others are also very common [5-7] and associated with a wide range of adverse physical and mental health outcomes, as well as drastically increased economic costs including both direct treatment costs (eg, ~US $100 million for off-base sleep care in 2012) as well as indirect costs such as increased accident risk and diminished military readiness [5-10].

There are well-recognized barriers to sleep medicine care within the US military health system (MHS). Most importantly, there is a gross shortage of trained specialist providers. Thus, high demand greatly exceeds the available supply. Further, traditional sleep interventions can be time- and resource-intensive, requiring multiple face-to-face treatment sessions that can be difficult to accommodate in military work schedules. Sleep telehealth approaches including telemedicine, internet or mobile health, and wearables represent promising potential solutions to help address the military’s sleep problems.

This paper presents preliminary results from a pilot phase of a larger, ongoing clinical implementation project funded by the US Department of Defense. We previously conducted focus groups and 1:1 interviews with diverse stakeholders including military patients, primary care managers (PCMs), and economic stakeholders to obtain an understanding of the overall sleep landscape in the US military [11]. Further, we created a novel sleep telehealth platform that includes a native mobile app and integrated commercial off-the-shelf (COTS) sleep tracker [12]. The objective of this study was to describe patient engagement and provider perceived effectiveness of a 10-day remote monitoring assessment in a pilot study. The purpose of the remote monitoring assessment was to provide evidence-based sleep treatment recommendations to patients and providers.

## Methods

### Study Design and Overview

Patients completed a comprehensive assessment via secure native mobile app, wore a COTS sleep tracker, completed daily sleep diaries, and completed brief symptom surveys via a smartphone twice during a 10-day intensive remote monitoring period (ie, 20 survey administrations across 10 days). Next, participants received an in-app personalized sleep assessment report. In parallel, an assessment report was generated for providers, which included provisional diagnoses and recommended next steps. Throughout this pilot study, participants continued with all aspects of routine clinical care. This paper presents salient details from this study.

### Participants

#### Patients With Sleep Problems

Participants were recruited from the Internal Medicine clinic and the Sleep Disorders Center at Walter Reed National Military Medical Center. Inclusion criteria included age between 18 and 75 years, being a military servicemember or Defense Enrollment Eligibility Enrollment System beneficiary, owning a smartphone, and having provider or self-referral for sleep problems. Exclusion criteria were pregnancy and untreated or uncontrolled (or both) medical or psychiatric illness.

#### Health Care Providers

Health care PCMs (including physicians, nurse practitioners, and physician assistants) were recruited via word of mouth from the Internal Medicine clinic at Walter Reed National Military Medical Center.

### Sleep Telehealth Platform

The sleep telehealth platform (WellTap) consists of a web-based portal for patients and providers, a native mobile app, and integrated wearable sensors using a COTS sleep tracker (in this study, Fitbit Inspire 2). As described elsewhere [11,12], the purposes of the overall platform are to (1) help PCMs assess sleep complaints and triage patients to the appropriate level of evidence-based care, (2) empower patients and PCMs to make evidence-based sleep treatment decisions, (3) deliver evidence-based behavioral sleep treatments via mobile devices, and (4) connect patients with sleep specialists in internet-based or physical sleep centers.

### Procedures

#### Recruitment and Study Onboarding

Following the onset of the COVID-19 pandemic, all research procedures were conducted remotely [13]. Potential participants were identified by health care providers or self-referred for having sleep problems and were contacted via telephone for eligibility screening. Interested volunteers were referred to a trained study coordinator who ensured informed consent, provided study instructions, and onboarded participants to the study app (downloaded from the Apple App store or Google Play store) using a secure code. Participants were provided Fitbit devices via priority courier. Study staff remained available to provide support or answer technical questions.

#### Sleep Assessment

**Figure 1:**
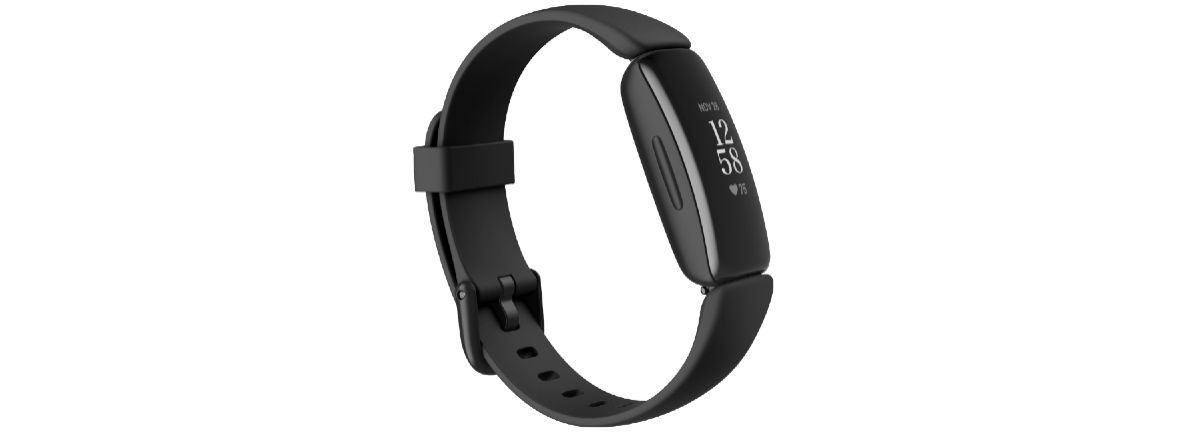
Participants wore a Fitbit Inspire 2 device throughout the study. Sleep data were captured in 1-minute epochs and integrated directly into the WellTap platform engine via an application programming interface. Variables including total sleep time, number of awakenings, wake after sleep onset, sleep efficiency, and sleep timing were included in personalized assessment results presented to patients in the app and to providers in provider reports.

The in-app sleep assessment included a sleep history questionnaire; several validated research questionnaires that are widely used within the Department of Defense to assess symptoms of obstructive sleep apnea (Berlin Questionnaire [14]), insomnia (Insomnia Severity Index [15]), depression (Patient Health Questionnaire-9 [16]), anxiety (Generalized Anxiety Disorder-7 [17]), posttraumatic stress disorder (Primary Care PTSD [posttraumatic stress disorder] Screen for DSM-5 [Diagnostic and Statistical Manual of Mental Disorders] [18]), chronic pain (Defense and Veterans Pain Rating Scale [19]), and history of traumatic brain injury (Brief Traumatic Brain Injury Screen [20]); standardized daily sleep diaries; and brief symptom surveys administered twice a day (morning and evening) that assessed mood, cognition, and energy level. Symptom surveys followed the format “*I feel* ___” followed by descriptors such as happy, sad, clear-headed, and tired. Responses ranged from “not at all” to “very” and were scored from 0 to 4, respectively. Participants also wore a Fitbit Inspire 2 device throughout the study (Figure 1). Sleep data were obtained in 1-minute epochs daily from the Fitbit device and integrated directly into the WellTap platform engine via an application programming interface. Total sleep time, number of awakenings, wake after sleep onset, sleep efficiency, and sleep timing were reported to patients in the app and to providers in provider reports.

#### Sleep Report

Following completion of the remote assessment, the platform generated separate sleep assessment reports for patients and PCMs. For patients, sleep assessment results were presented in the app (Table 1). For providers, sleep assessment results were provided (via store and forward) in an editable electronic document format (eg, PDF or DOCX [or both]; Table 1). The content for both reports were derived from the 10-day continuous sleep monitoring assessment and tailored for patients or providers on the basis of feedback gained in an earlier qualitative phase of this study [11,12].

Patients with sleep problems received their personalized sleep assessment results in the app, including integrated results from both patient-reported and COTS wearable data. Provider reports were provided in an editable electronic document format.

**Table 1:** Patient and provider assessment report content.

Patient Report (in-app)		Provider Report	
**Sleep assessment results**		**Summary**	
	Description of possible diagnoses		Population health risks
	Key comorbidities		Likely sleep diagnoses
	Sleep habits: schedule		Key comorbidities to consider
	Sleep habits: presleep routine		Home sleep apnea test eligible?
	Sleep habits: environment		Patient preferences and motivation
	Sleep habits: sleep thinking		Recommendations
**Sleep and wearable device**		**History of present illness**	
	Sleep diary	**Review of systems**	
	Wearable results		Sleep apnea
**Recommendations**			Circadian rhythm sleep disorder or shift work
**Fitbit report**			Restless legs syndrome
	Total sleep time		Parasomnias
	Sleep details		Narcolepsy
**Sleep diary**			Insomnia
	Total sleep time	**Psychiatric and daytime sequelae**	
	Sleep details	**Past medical history**	
			Past medical conditions
			Past surgical history
			Current medications
			Medication allergies
		**Social history**	
		**Family history**	
		**Questionnaire results**	
		**Self-reported sleep parameters**	
			Sleep monitoring
			Sleep diary results
			Wearable results
			Interpretation of discrepancies between sleep diary and wearable device
		**Health sleep habits summary**	
			Sleep schedule and routine
			Bedroom environment
			Sleep beliefs
			Other lifestyle factors

### Outcome Measures

#### Patient Engagement

Patient engagement was defined a priori based on behavioral adherence metrics including (1) completion of the baseline assessment, (2) wearing the COTS sleep tracker, (3) completion of daily sleep diaries, (4) completion of daily symptom surveys (ie, EMA assessments), and (5) review of the personalized sleep report. In addition, following the 10-day remote monitoring assessment, patients completed a brief survey to assess perceived ease of completion. Individual items asked, “How easy was it for you to complete the assessment questions on your device?” and “How easy was it to navigate the assessment on your device?” Five possible responses ranged from “Not at all” to “Very.” A third question was asked about the length of assessment questions; responses ranged from “too short” to “good” to “too long” on a 5-point bipolar response scale. Finally, participants were asked (yes/no) if they needed human support to complete the remote monitoring assessment, as well as willingness to consider future sleep telehealth approaches (yes/no).

#### Provider Effectiveness

Effectiveness was defined a priori as usability, acceptability, perceived improvement, perceived credibility, and overall satisfaction. After reviewing sample assessment reports, providers completed an anonymous 16-item questionnaire developed for this study. All responses were presented on a 5-point scale ranging from “Not at all” to “Very” and were scored from 0 to 4, respectively. In addition, providers were asked, “How likely are you to recommend patients into this study?” Responses again ranged from “Not at all” to “Very.”

### Ethical Considerations

This study was approved by the institutional review board at Walter Reed National Military Center (WRNMMC-2019-0258). All patients with sleep problems provided written informed consent. PCMs were not required to provide informed consent to complete the anonymous survey. Patients with sleep problems were compensated up to US $65 for participation in the study. PCMs were thanked for their participation.

## Results

### Participants

Between March 10 and October 20, 2021, a total of 35 patients (57.1% female, mean age 45.7, SD 15.7 years) completed the 10-day assessment. The majority self-identified as White (45.7%) or Black (31.4%), and more than half of them (51.4%) were active-duty military personnel. Table 2 presents demographic and clinical characteristics. In addition, 24 PCMs reviewed reports and completed the anonymous effectiveness survey.

**Table 2:** Participant demographics (N=35).

Characteristics			Value
Age (years), mean (SD)			45.7 (15.7)
**Sex, n (%)**			
	Male	15 (42.9)	
	Female	20 (57.1)	
**Race and ethnicity, n (%) **			
	White	16 (45.7)	
	Black	11 (31.4)	
	Hispanic or Latino	5 (14.3)	
	Asian	2 (5.7)	
	Native American or Alaskan Native	1 (2.9)	
**Military status, n (%) **			
	Active-duty military	18 (51.4)	
	Retired military	9 (25.7)	
	Civilian	8 (22.9)	
**Military rank (n=18; enlisted pay grade^a^), n (%)**			
	Enlisted personnel 3	3 (16.7)	
	Enlisted personnel 4	2 (11)	
	Enlisted personnel 5	3 (16.7)	
	Enlisted personnel 6	1 (5.6)	
	Enlisted personnel 7	2 (11)	
	Commissioned officer 1	1 (5.6)	
	Commissioned officer 4	2 (11)	
	Commissioned officer 5	3 (16.7)	
	Warrant officer 4	1 (5.6)	

^a^Within each of these categories, higher numbers represent higher rank and corresponding higher annual compensation.

### Patient Engagement

There were no dropouts in this study. All participants completed the baseline assessment, wore the COTS sleep tracker, reviewed their in-app sleep report, and completed the postassessment survey, reflecting 100% behavioral adherence for these metrics. In terms of daily sleep diaries and symptom surveys, on average, participants completed 95.1% (range 70%-100%) of daily sleep diaries and 95.3% (range 80%-100%) of daily EMA surveys (Table 3). In terms of perceived ease of use, nearly all (97.2%) participants reported that it was “mostly easy” or “very easy” to complete the remote monitoring assessments and “mostly easy” or “very easy” to navigate the assessment on their devices. Overall, 91.4% of participants reported that the length of questions was “good” (ie, neither too short nor too long) and that they did not need human support during the remote assessment. All participants reported that they would consider sleep telehealth in the future.

**Table 3:** Summary of patient engagement (N=35).

Patient engagement^a^	Values
Completed the baseline assessment, n (%)	35 (100)
Wore the commercial off-the-shelf (COTS) sleep tracker, n (%)	35 (100)
Completed daily sleep diaries, mean (SD); n (%); range	9.5 (0.7); 10 (95); 7-10
Completed daily ecological momentary assessments (EMAs), mean (SD); n (%); range	19.1 (1.2); 20 (95.5); 16-20
Reviewed sleep report, n (%)	35 (100)

^a^Patient engagement was defined a priori based on key metrics of behavioral adherence.

### Provider Effectiveness

Usability, acceptability, perceived improvement, perceived credibility, and overall satisfaction of the sleep assessment report were highly rated. For instance, in terms of usability, 100% of providers reported that the sleep report was “mostly” or “very” usable for assessing sleep problems, 95.6% of them reported that the sleep report was “mostly” or “very” usable for providing evidence-based-sleep recommendations, and 100% of them reported that the sleep report was “mostly” or “very” usable for documenting sleep problems. Most providers (86.4%) reported that they would be “likely” or “very likely” to refer patients to the study. Results are presented in Table 4.

**Table 4:** Summary of provider effectiveness outcomes (n=24).

			Very not, %		Not, %		Neutral, %		Term, %		Very, %
**Usability**											
	How usable is the sleep report for assessing sleep problems among patients you see?	0		0		0		56.5		43.5	
	How usable is the sleep report for providing evidence-based treatment recommendations (treatment planning)?	0		0		4.4		65.2		30.4	
	How usable is the sleep report for documenting sleep problems?	0		0		0		45.5		54.6	
**Acceptability**											
	How acceptable would it be for the hospital (or DoD) to adopt this sleep telemedicine platform throughout the system?	0		0		4.8		52.4		42.9	
**Perceived improvement**											
	How much would the sleep report improve your assessment of sleep problems?	0		0		8.7		56.5		34.8	
	How much would the sleep report improve your evidence-based sleep treatment recommendations (treatment planning)?	0		0		13.0		47.8		39.1	
	How much would the sleep report improve your documentation of sleep problems?	0		0		4.4		52.2		43.5	
**Credibility**											
	How credible to you are the sleep assessment results in the sleep report?	0		0		17.4		69.6		13.0	
	How credible to you are the evidence-based sleep treatment recommendations in the sleep report?	0		0		8.7		69.6		21.7	
**Overall satisfaction**											
	Overall, how satisfied are you with the content of the report?	0		0		0		69.6		30.4	
	Overall, how satisfied are you with the format of the report?	0		0		9.1		63.6		27.3	
	Overall, how satisfied are you with the length of the report?	0		9.1		27.3		40.9		22.7	

## Discussion

Sleep problems are common and costly in the US military, and demand for sleep specialty care far exceeds the available supply. In non-MHS samples, remote diagnosis and treatment of sleep disorders is a viable approach to increase access to evidence-based care, including, for example, remote diagnostic testing for obstructive sleep apnea [21,22] and mobile delivery of cognitive-behavioral sleep treatments [23,24]. Results from this pilot study strongly support telehealth approaches as a potential pathway to increase access to evidence-based sleep medicine care in the US military.

Using a novel sleep telehealth platform that incorporates patient-reported as well as passively gathered objective data, patients demonstrated high levels of engagement during a 10-day intensive remote monitoring assessment. All participants achieved key behavioral adherence metrics, having completed the baseline assessment, reviewed their personalized sleep assessment results in-app, and completed the postassessment survey. Further, adherence with daily sleep diaries and symptom surveys was notably high (exceeding 95% of survey completion), and patients reported high levels of satisfaction with the intensive remote monitoring assessment. In addition, nonspecialist health care providers found the novel sleep assessment report usable and likely to improve evidence-based clinical decision-making as well as documentation.

In the MHS as well as in the broader health care universe, sleep telehealth approaches such as the one in this study can help to streamline consult efficiency while also helping PCMs to assess sleep complaints and triage patients to the appropriate level of evidence-based care, including specialist consultations when indicated. Such an approach will empower patients and PCMs to make evidence-based sleep treatment decisions and increase access to care. If proven successful in larger studies, a sleep telehealth platform and remote monitoring holds great promise to increase patient and provider engagement in support of improved patient, health system, and military-relevant outcomes. Such research efforts are currently underway.

## Abbreviations

COTS: commercial off-the-shelf
DSM-5: Diagnostic and Statistical Manual of Mental Disorders
MHS: military health system
PCM: primary care manager
PTSD: posttraumatic stress disorder

## Data Availability

Consistent with the policies of Human Research Protections Program in the Department of Research Programs at Walter Reed National Military Medical Center, any request for raw data will require a data sharing agreement (and protocol modification, if applicable) to limit the use of data and to protect participant confidentiality. Any recipient of a limited, deidentified data set will be prohibited from identifying or reidentifying any participant whose data are provided.
